# Complete mitochondrial genome and microsatellite marker development of the Antarctic scallop (*Adamussium colbecki*) for its population genetics analysis

**DOI:** 10.1371/journal.pone.0351123

**Published:** 2026-06-15

**Authors:** Hye Jin Park, Soon Young Hwang, Hee-kyu Choi, Seo Yeon Byeon, Young Wook Ko, Sang Rul Park, Sanghee Kim, Hyuk Je Lee

**Affiliations:** 1 Molecular Ecology and Evolution Laboratory, Department of Biological Science, College of Life and Environment, Sangji University, Wonju, Korea; 2 Oceanic Climate and Ecology Research Division, National Institute of Fisheries Science, Busan, Korea; 3 Division of Life Sciences, Korea Polar Research Institute, Incheon, Korea; 4 Estuarine and Coastal Ecology Laboratory, Department of Marine Life Sciences, Jeju National University, Jeju, Korea; National Cheng Kung University, TAIWAN

## Abstract

*Adamussium colbecki* is an ecologically important species (class Bivalvia) endemic to the Southern Ocean, but genomic resource for this species has remained yet limited. In this study, we first report the complete and annotated mitochondrial genome of *A. colbecki*, assembled using MGI paired-end sequencing. The circular mitogenome was 15,269 bp in length and comprised of 12 protein-coding genes (PCGs), two rRNA genes, and 18 tRNAs, with the *atp8* gene absent, as commonly observed in bivalve molluscs. Phylogenetic analysis based on 11 mitochondrial PCGs (7,053 bp) revealed that *A. colbecki* formed a distinct and well-supported lineage within Pectinidae, the most closely related to *Placopecten magellanicus*. The 12S and 16S rRNA regions of the mitogenome were analyzed as mitochondrial DNA markers. In addition, using whole nuclear genomic information 144,512 perfect microsatellite loci (SSRs) were identified, and 30 primer-pairs were designed, among which seven polymorphic markers were developed. We then tested for their applicability to population genetics analysis and found that they were relatively highly polymorphic. These newly generated genomic resources will provide versatile genetic markers for future studies on population structure, genetic diversity, and evolutionary (demographic) history of *A. colbecki*, contributing to long-term population monitoring and conservation in the Antarctic marine ecosystem.

## Introduction

Genetic diversity is a key component of biodiversity that enables species to adapt to environmental changes, and helps to maintain community resilience under environmental stresses [1]. Loss of genetic diversity is suggested to be directly associated with reduced population viability and an increased risk of extinction, particularly in small or isolated populations [2,3]. Recognizing these threats, global policy frameworks have increasingly emphasized the need to conserve genetic variation both within and between species. The Kunming–Montreal global biodiversity framework, adopted under the convention on biological diversity (CBD) in 2022, set clear targets for safeguarding at least 90% of genetic diversity within all species by 2030, highlighting the urgency of genomic data generation and monitoring across taxonomic groups [4]. Similarly, the Nagoya protocol, adopted under the CBD, provides a legal foundation for access to genetic resources and the fair and equitable sharing of benefits arising from their utilization, thereby reinforcing the importance of preserving genetic material as a global common [5]. Moreover, the United Nations sustainable development goals (SDGs) explicitly address the conservation of genetic diversity in marine and terrestrial ecosystems as essential for long-term ecological sustainability [6]. Despite these international efforts, the genetic diversity and population structure of many non-model species‒particularly those in polar ecosystems‒remain relatively poorly characterized, due to challenges in specimen collection, limited research efforts and a lack of tailored molecular tools [7,8].

The Antarctic scallop, *Adamussium colbecki* (Smith, 1902) is a benthic bivalve species endemic to the Southern Ocean, with a wide distribution along the coasts of the Ross Sea and Terra Nova Bay [9]. This species inhabits a range of diverse environmental conditions, from deep ice-free waters to shallow regions beneath sea ice, and plays a crucial role in nutrient cycling, energy transfer, and the structuring of benthic food webs [10–13]. *Adamussium colbecki* exhibits an annual reproductive cycle, with spawning occurring in the austral spring, followed by the development of planktotrophic larvae [14,15]. As adults, individuals adopt a sessile lifestyle, settling on and attaching to substrates [14]. Lifespan estimates vary considerably, ranging from approximately six to over 19 years, depending on habitat conditions [10,14]. Recent studies have shown that *A. colbecki* remains ice-free in environments where sub-surface ice formation is common, suggesting a unique adaptation to cryofouling avoidance [16]. Due to its sensitivity to environmental change and its ecological importance*, A. colbecki* is considered a sentinel species for monitoring the impacts of climate change and anthropogenic disturbances in Antarctic marine ecosystems [13,17,18]. In this context, accumulating evidence suggests that ongoing environmental changes may influence important biological processes in this species. A recent study has reported that environmentally driven reduced growth rates in juvenile *A. colbecki* can negatively affect individual fitness and, in turn, compromise long-term population sustainability in *A. colbecki* [19].

Despite its ecological significance, genetic or genomic research for this species remain scarce, with most studies focused on ecological aspects of the species. A previous study analyzed the 18S rDNA sequence of *A. colbecki* to investigate its phylogenetic relationships with other bivalves, providing the first molecular insights; however, the scope was limited to a single nuclear ribosomal gene and its taxonomic placement was not yet fully resolved [20]. Nevertheless, a more recent study generated the first transcriptomic dataset for this species as a part of genomic research efforts, providing an important foundation for future molecular and ecological studies [21]. However, this transcriptome-based approach was restricted to expressed gene regions, while complete mitochondrial genome or any population-level informative genetic markers, such as microsatellites, mitochondrial DNA (mtDNA) and nuclear DNA (nuDNA) single nucleotide polymorphisms (SNPs), might be needed for its population genetic and phylogeographic studies.

In this study, we aimed to provide the molecular resources available for *A. colbecki* by conducting both mitochondrial and nuclear genomic analyses. The specific objectives were: (1) to construct and annotate the complete mitochondrial genome of *A. colbecki*; and (2) to determine the phylogenetic position of *A. colbecki* within family Pectinidae and (3) to develop mtDNA and microsatellite markers and conduct preliminary validation of newly developed genetic markers. These obtained genetic/genomic resources will facilitate future research on genetic diversity, population structure, and also long-term monitoring of this ecologically and biogeographically important Antarctic scallop. Furthermore, the findings will inform on effective conservation and management strategies for *A. colbecki* in the context of ongoing climate-driven environmental change in the Southern Ocean.

## Materials and methods

### Sample collection and DNA extraction

**Fig 1:**
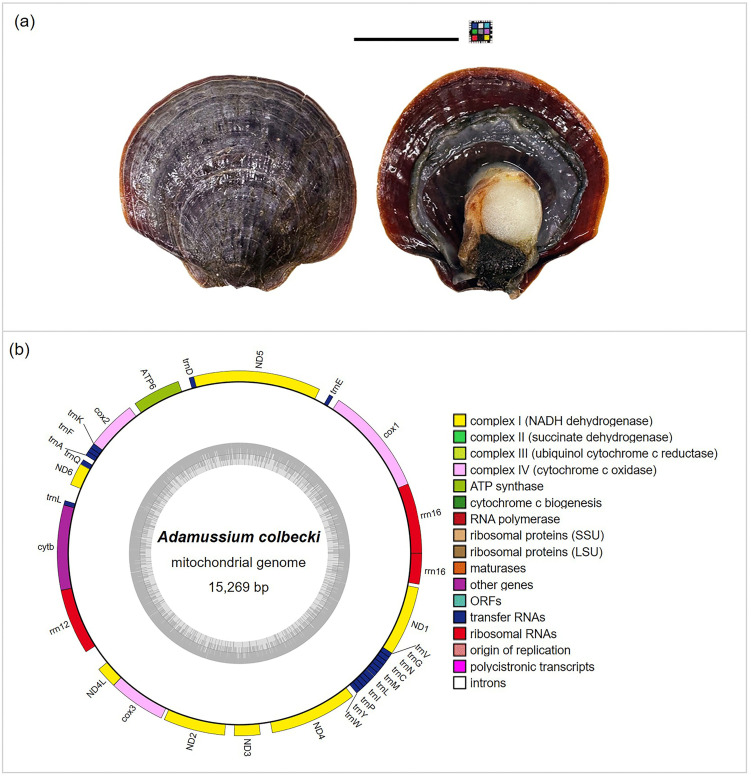
External morphology of *Adamussium colbecki.* **(a)** External morphology of *A. colbecki* (scale bar = 5 cm). **(b)** Circular map of the complete mitochondrial genome (15,269 bp). Genes are color-coded by functional category, as shown in the legend. The inner grey ring represents GC content across the mitogenome.

A total of 153 samples were collected approximately 0.6 km from the Jang Bogo Antarctic Research Station (74◦37′27ʺ S, 164◦13′54ʺ E) in Terra Nova Bay between 2018 and 2019. This study did not require for ethical review or approval for animal research because the study species (*A. colbecki*) is not listed as an endangered or protected species. Field research in the study area was conducted with permission from the Ministry of Oceans and Fisheries (MOF) of the Republic of Korea. For genetic analyses, a small portion of adductor muscle tissue (approximately 5 × 5 mm) was excised from each individual (Fig 1). The tissue samples were immediately preserved in 1.5 ml micro-centrifuge tubes containing 99% ethanol and stored at 4 °C until DNA extraction. Genomic DNA was extracted from the muscle tissue of *A. colbecki* using a DNeasy Blood and Tissue Kit (Qiagen, Germany), according to the manufacturer’s instructions. The extracted DNA was concentrated and purified, and its concentration was measured using a NanoDrop One spectrophotometer (Thermo Scientific, USA). The purified DNA was then stored at −20 °C until use.

### Ethics statement

Ethical review and approval for animal research were not required for this study because *A. colbecki* is a species commonly harvested through commercial fishery activities and is not listed as an endangered or protected species. In addition, field research in this area was approved by the Ministry of Foreign Affairs of the Republic of Korea.

### Next generation sequencing (NGS) and mitochondrial genome assembly

Genomic DNA extracted from *A. colbecki* was fragmented using the S220 Ultrasonicator (Covaris, USA), and sequencing libraries were prepared using the MGIEasy DNA Library Prep Kit (MGI, China). The NGS sequencing library was prepared from genomic DNA extracted from a single individual of *A. colbecki*. Paired-end sequencing (150 bp) was performed on the MGIseq platform. The sequencing generated a total of 239,519,456 reads, corresponding to 35,927,918,400 bp of raw data. The mitochondrial genome sequences were extracted from the extensive dataset generated for microsatellite marker development, and mitochondrial genome assembly was subsequently performed. Low-quality bases and sequencing artifacts were removed using Trimmomatic v0.39 in paired-end mode. The mitochondrial genome was assembled using GetOrganelle v1.7.7 with multiple k-mer values (21–105). Genome annotation and visualization were subsequently performed using MitoZ v2.3. Gene annotation was further refined using Geneious Prime^®^ (Biomatters Ltd., New Zealand), with reference to previously reported mitochondrial genomes.

### Mitochondrial DNA marker development

Ribosomal RNA (12S and 16S) regions were identified from the annotated mitochondrial genome of *A. colbecki*. Primer sets targeting these regions were designed using Geneious Prime^®^ (Biomatters Ltd., New Zealand). These regions were specifically focused because partial 12S and 16S rRNA gene sequences of *A. colbecki* are previously available from GenBank (e.g., accession nos. HM600759.1 [22] and HM600752.1 [22], EU379437.1 [23]), and they have been commonly used in earlier phylogenetic analyses, and thus, developing primers for these loci enable comparability with previous phylogenetic studies [20]. PCR amplifications were performed under standardized conditions, with an initial denaturation at 94 °C for 2 minutes, followed by 35 cycles of denaturation at 94 °C for 30 seconds, annealing at 58 °C for 30 seconds, and extension at 72 °C for 1 minute, with a final extension at 72 °C for 10 minutes. Amplified PCR products were visualized by electrophoresis on 2% agarose gels stained with RedSafe (iNtRON Biotechnology, Korea). Successfully amplified products were purified using Exonuclease I and Shrimp Alkaline Phosphatase treatments, and subsequently sequenced on an ABI 3730xl DNA Analyzer (Applied Biosystems, USA).

MtDNA diversity statistics based on concatenated sequences of 12S and 16S regions (1,277 bp), such as number of haplotypes (*N*_*H*_), haplotype diversity (*h*), and nucleotide diversity (*π*) were estimated for only 16 individuals using Arlequin v3.5 [24]

### Microsatellite marker development

Microsatellite markers were identified from the genomic sequence data using the MGIseq platform output. SSR detection was performed on assembled genomic sequences (contigs) of *A. colbecki* using Krait v1.3.3 [25], considering only perfect tandom repeats (i.e., uninterrupted repeat motifs). The threshold of minimum repeat numbers was set to mononucleotide ≥12 repeats, dinucleotide ≥7 repeats, trinucleotide ≥5 repeats, tetranucleotide ≥4 repeats, pentanucleotide ≥4 repeats, and hexanucleotide ≥4 repeats [26]. These thresholds were used to define candidate microsatellite loci and exclude very short repeat regions. Motif standardization was conducted using Level 3, which groups equivalent motifs by considering complement and rotational variations [25]. A total of 239,519,456 reads corresponding to 35,927,918,400 bp were analyzed. In total, 144,512 perfect simple sequence repeats (SSRs) were identified, including di-nucleotide (37,544 loci, 25.98%), tri-nucleotide (25,359 loci, 17.55%), tetra-nucleotide (15,410 loci, 10.66%), and penta- and hexa-nucleotide motifs (4,809 loci, 3.33%). Among SSRs with a single predicted amplicon, 30 candidate microsatellite loci were first selected based on repeat characteristics and primer design suitability (S1 Table). PCR products were evaluated on 2% agarose gels stained with RedSafe and among them, the 10 loci that showed clear amplification in at least six individuals, were selected for fragment analysis. Of these, the forward primers of only seven loci were synthesized with fluorescent dyes (FAM or HEX) and used for capillary electrophoresis on an ABI 3730xl DNA Analyzer (Applied Biosystems). Fragment sizes were analyzed using GENEMAPPER v5.0 software with ROX 500 as a size standard.

Diversity statistics at the seven microsatellites, including observed number of alleles (*N*_*A*_), expected heterozygosity (*H*_*E*_), observed heterozygosity (*H*_*O*_), polymorphic information content (PIC; [27]), inbreeding coefficient (*F*_*IS*_) and Hardy–Weinberg Equilibrium (HWE) deviations were calculated using GENEPOP 4.0 [28] and FSTAT 2.9.3.2 [29]. Deviation from HWE at each locus was assessed using *F*_IS_ values in 27–30 individuals [30]. The 95% significance level for HWE test was adjusted using a Bonferroni correction. The frequencies of null alleles were estimated across the seven loci to test for the reliability of microsatellite genotyping using both MICRO-CHECKER v2.2.3 [31] with 1,000 randomizations at 95% confidence level and GENEPOP. Linkage disequilibrium (LD) among all pairs of loci was also tested using GENEPOP, and significance levels were adjusted using a Bonferroni correction.

### Mitogenome-based phylogenetic analysis

To determine the phylogenetic placement of *A. colbecki* within the family Pectinidae and to provide a comparative framework for interpreting its mitogenome characteristics, a phylogenetic analysis was conducted using 11 mitochondrial protein-coding genes (PCGs); *cox1–3*, *cytb*, *nad1–6*, *nad4L*), resulting in a concatenated alignment of 7,053 bp. Homologous gene sequences from 12 other Pectinidae species and two taxa (*Mytilus trossulus* and *Brachidontes exustus*, GenBank accession Nos. NC_007687 and NC_024882) were used as outgroup [32,33]. Sequence alignment for each gene was performed using a multiple alignment tool in Geneious Prime^®^ (Biomatters Ltd., New Zealand) with default parameters, and the aligned sequences were concatenated into a single dataset. For maximum likelihood (ML) analysis, the best-fit substitution model (GTR + I + G) was determined using jModelTest v2.1.10 based on the Akaike Information Criterion (AIC). The ML tree construction was conducted with IQ-TREE v2.4.0 [34], and statistical node support was estimated using ultrafast bootstrap (UFBS) with 1,000 replicates [35]. The resulting tree was visualized in a rooted format using FigTree v1.4.4. In parallel, a neighbor-joining (NJ) tree was constructed using the NJ method implemented in MEGA v12, based on the Tamura–Nei distance model [36]. The analysis included only coding positions, with pairwise deletion of ambiguous sites. Bootstrap support was calculated from 1,000 replicates [37], and the optimal tree with the lowest total branch length (2.044) was selected. Both trees showed consistent topologies, supporting the distinct phylogenetic position of *A. colbecki* within family Pectinidae.

## Results

### The complete mitochondrial genome mapping and mtDNA marker development

A total of 239,519,456 paired-end reads (150 bp) were generated using the MGISEQ 2000 platform, yielding approximately 35.93 Gb of raw data. After quality control and filtering, de novo assembly of the sequencing reads produced 875,673 contigs, with a total assembled genome size of 963,461,770 bp. The assembly had an N50 value of 1,961 bp, N80 of 648 bp, and N90 of 428 bp. The longest scaffold was 45,733 bp, and the shortest was 200 bp. The GC content of the assembled genome was 36.88%, and the average contig length was 1,100 bp (Table 1).

**Table 1:** Summary statistics of generated read sequence data and assembly of *Adamussium colbecki* genome.

Sequencing Data Summary	
Platform	MGISEQ 2000
Library Type	Paired-end
Read Length	150
# of Reads	239,519,456
Total bp	35,927,918,400
Assembled Genome Summary	
Number of Contigs	875,673
N50	1961
N80	648
N90	428
Longest (Shortest) scaffolds bp	45,733 (200)
GC level	36.88%
Total Assembled bp	963,461,770
Average length	1100.25

The final assembled mitochondrial genome was a circular DNA molecule of 15,269 bp in length, with a GC content of 45.34% and no ambiguous bases (N content = 0). Gene annotation revealed that the circular mitochondrial genome contains 12 PCGs, two rRNA genes (12S and 16S rRNA), and 18 transfer RNA genes (tRNAs). The identified PCGs included typical mitochondrial genes such as *cox1*, *cyt b*, *nad1–6*, *nad4L*, *atp6*, and *cox3*, which are consistent with those found in other scallop mitogenomes. Three tRNA genes (tRNA-Arg, tRNA-His, and tRNA-Ser) were not detected by the annotation pipeline, and *atp8* gene was also not found from the present assembly. All genes are encoded in strand-specific orientation across the circular genome (Fig 1). Functional categorization of annotated genes is illustrated in the circular genome map, where genes are color-coded according to their roles in mitochondrial respiratory complexes, ATP synthesis, ribosomal function, and tRNA transport. The innermost grey ring represents GC content, indicating a relatively uniform distribution of GC content across the mitogenome.

The primer design for 12S and 16S rRNA genes was guided by annotated regions in the newly assembled mitochondrial genome. A total of four primer sets were generated for each locus, with expected amplicon sizes ranging from 700–712 bp (Table 2). Only four haplotypes were identified with *h* value of 0.35 and π of 0.0004 from 16 samples. These mtDNA markers can provide a basis for future population genetic studies and species-level identification of *A. colbecki*.

**Table 2:** Primer information for 12S rRNA and 16S rRNA gene markers developed based on the complete mitochondrial genome of *Adamussium colbecki*.

Locus	Primer name	Sequences (5′-3′)	Length (bp)
12S rRNA	ACI_12S_183FW	GGTTTAAACCCTGAGCGTCAG	700
	ACI_12S_183RV	GGGCGCACCTTCCAGTAC	
	ACI_12S_166FW	CATGACTCCAGTGGAAAGGT	712
	ACI_12S_166RV	CACCTTCCAGTACCCCTACC	
16S rRNA	ACI_16S_181FW	ATGAGTAGTCTGAGGTTTAGTGG	711
	ACI_16S_181RV	AAAATAGGAAACCTAGGCCCG	
	ACI_16S_251FW	AGTTAAAGGAACTCGGCAAATAGA	700
	ACI_16S_251RV	TTAGCCATTAGCCACCGAGAC	

### Phylogenetic relationships

**Fig 2:**
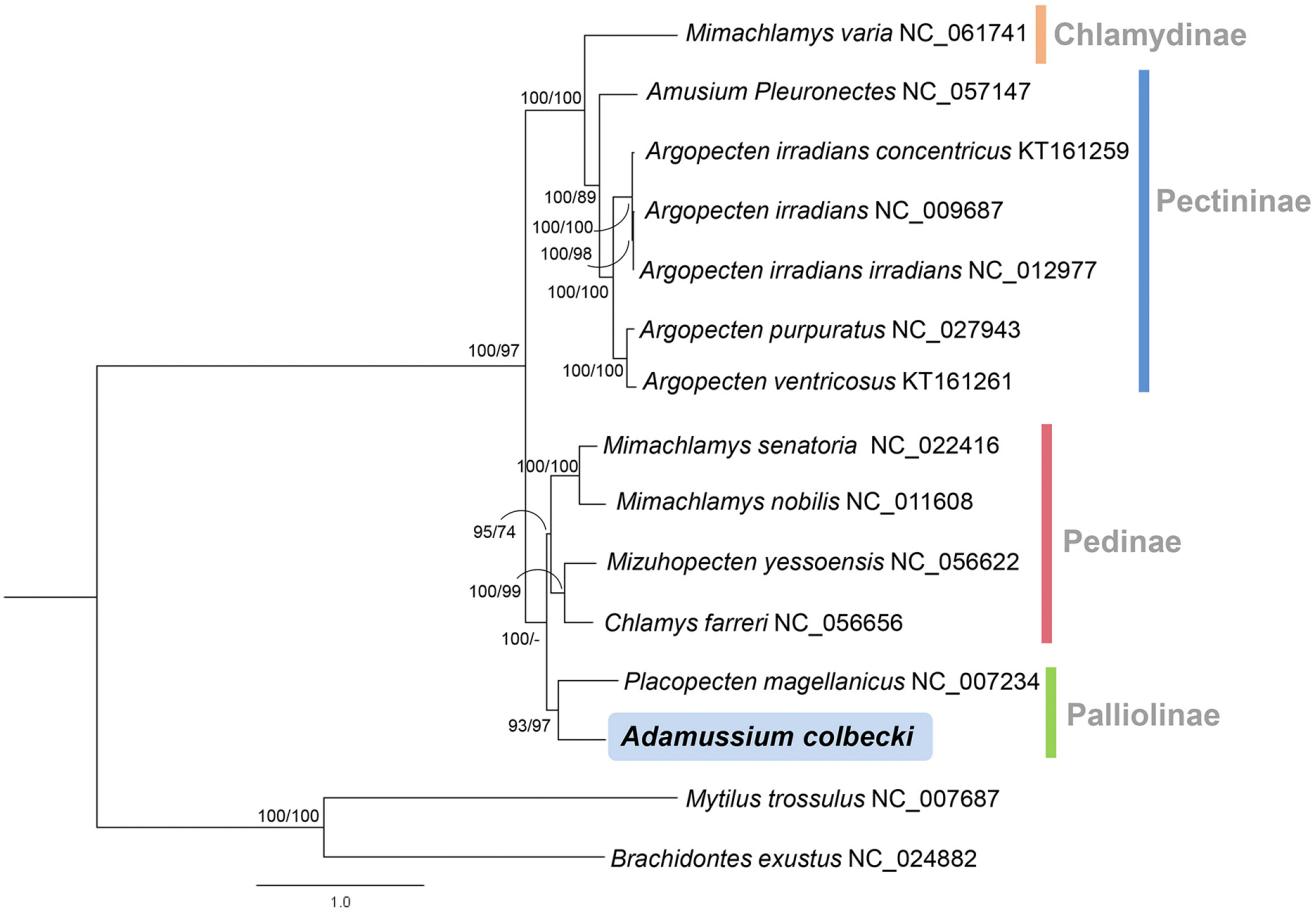
Molecular phylogenetic tree of 13 Pectinidae species including *Adamussium colbecki.* Neighbor-joining (NJ) and maximum likelihood (ML) trees were reconstructed based on 11 mitochondrial PCGs (7,053 bp). The tree was rooted with two outgroup taxa (*M. trossulus* and *B. exustus*). Numbers at branches indicate bootstrap support values for NJ (left) and ML (right). Branching patterns and lengths follow the NJ analysis.

Phylogenetic analysis based on 11 mitochondrial PCGs (totaling 7,053 bp) was conducted with two taxa (*M. trossulus* and *B. exustus*) as an outgroup. The resulting phylogeny revealed two major clades within the family Pectinidae. One clade comprised *Mimachlamys varia*, *Amusium pleuronectes*, and species of the genus *Argopecten*, forming a strongly supported monophyletic group (bootstrap = 100/100). The second clade included *Mimachlamys senatoria*, *Mimachlamys nobilis*, *Mizuhopecten yessoensis*, *Chlamys farreri*, *Placopecten magellanicus*, and *Adamussium colbecki*. Within this clade, *A. colbecki* was recovered as the sister species to *P. magellanicus*, with strong bootstrap support (93/97; Fig 2).

### Microsatellite marker development

A total of 144,512 perfect SSR loci were identified from the assembled genome of *A. colbecki*, comprising six motif types ranging from mono- to hexa- nucleotide repeats (Table 3). Mononucleotide repeats were the most abundant, accounting for 61,390 loci (42.5%), followed by dinucleotide (37,544 loci, 26.0%) and trinucleotide repeats (25,359 loci, 17.6%). The average repeat length ranged from 15.51 bp (mono) to 26.99 bp (hexa), and the overall relative abundance and density of SSRs were 63.72 loci/Mb and 988.47 bp/Mb, respectively, for mononucleotides. Among the 144,512 SSR loci, 17,323 candidates with a single predicted amplicon were prioritized, and 30 primer pairs were initially designed (S1 Table). PCR results showed clear and reproducible amplification for 10 loci in at least six individuals. These 10 loci were selected for fluorescent labeling (FAM or HEX) and further fragment analysis. However, fragment genotyping reliably yielded allele profiles only for seven microsatellite loci across 27–30 individuals. *N*_*A*_ ranged from 13 to 19, with a mean of 15.3, indicating high allelic diversity (Table 3). The *H*_*O*_ ranged from 0.48 to 0.80, while *H*_*E*_ values ranged from 0.83 to 0.94, suggesting substantial genetic variation among individuals. Four loci (ACI-17, ACI-19, ACI-21, ACI-27) significantly deviated from HWE (*P* < 0.05), whereas the remaining loci showed non-significant deviation. The *F*_IS_ values ranged from 0.11 to 0.45 and *PIC* values from 0.79 to 0.91. No consistent evidence of potential genotyping errors due to stuttering or large allele dropout was detected across the loci. Estimated null allele frequencies based on the previous method [38] ranged from 0.0785 (ACI23) to 0.2470 (ACI19) (mean = 0.1264). These values fall within ranges commonly reported for polymorphic microsatellite markers and are unlikely to affect results of downstream population-level analyses [39]. These results were consistent with estimates obtained using GENEPOP, which indicated null allele frequencies ranging from 0.0000 (ACI23) to 0.2947 (ACI19) (mean = 0.133) across the seven loci.

**Table 3:** Summary statistics for seven validated microsatellite markers developed for *Adamussium colbecki*. For each locus, the number of individuals genotyped (*N*), number of alleles (*N*_*A*_), observed heterozygosity (*H*_*O*_), expected heterozygosity (*H*_*E*_), inbreeding coefficient (*F*_IS_), polymorphic information content (*PIC*), and Hardy–Weinberg equilibrium P-values are shown.

Loci	Primer sequence (5′- 3′)	Motif	repeat	Ta (°C)	*N*	*N*_*A*_	*H*_*O*_	*H*_*E*_	*F*_IS_	*PIC*	HWE *P*-values
ACI-17											
F	GAGTACCTTGTTAAATCGG	GTT	12	50.57	29	17	0.7241	0.9365	0.230	0.9147	0.0163
R	GATTCGATGACATGTTCC			50.58							
ACI-19											
F	ACAAGAAGAAACAGGAGG	AGG	12	51.22	27	13	0.4815	0.8742	0.454	0.8440	< 0.0001
R	AAGGACAGATACATGTAGG			50.57							
ACI-21											
F	ATGTGTCACTCATGTACC	CA	17	51.14	28	19	0.6786	0.9338	0.277	0.9114	< 0.0001
R	TTGAACTATGTATAGGGACC			51.03							
ACI-22											
F	CGGTAGTCCTTTATTTGG	GAA	11	49.66	28	13	0.7500	0.8870	0.157	0.8578	0.0833
R	GAATACGAGTGTTACTACC			49.72							
ACI-23											
F	CTTCATCATGACATCATCG	ATC	11	51.17	30	10	0.7333	0.8305	0.119	0.7938	0.3652
R	GGTGATTATATATGACGAGG			50.04							
ACI-27											
F	GTACACAAAAATACCCTCC	AAG	11	50.68	29	20	0.7241	0.9304	0.225	0.9083	< 0.0001
R	TGTAGCCAATGTGTTACC			51.77							
ACI-30											
F	GGATCCACTCTTTTTAGC	CT	17	49.95	30	15	0.8000	0.8932	0.106	0.8674	0.6390
R	TATAGAGTACTGTAGTGTCG			50.38							
Mean					–	15.3	0.6988	0.8979	0.225	0.8711	

## Discussion

To our best knowledge, this study is the first to address the whole mitogenomic features of *A. colbecki*, which is an ecologically valuable species in the Antarctic benthic ecosystem, particularly concerning the trophic web dynamics and the community stability [40]. The microsatellite as well as mtDNA markers developed in this study can serve as useful tools in future research for an understanding of genetic diversity and population structure of the Antarctic scallop. The constructed circular mitochondrial genome consisted of 15,269 bp and exhibits the typical gene composition of scallop mitogenomes, including 12 PCGs, two rRNA genes, and 18 tRNAs. Notably, the *atp8* gene was not detected in the present assembly, a pattern that has been frequently reported in bivalve mitochondrial genomes [41,42]. In addition, recent studies have shown that *atp8* is extremely short and highly variable in Pectinidae, and has frequently been identified in mitogenomes where it was previously thought to be absent, suggesting that non-detection may reflect annotation challenges rather than true gene loss [43]. However, to evaluate whether the *atp8* gene was present but undetected due to low sequence similarity, we analyzed five closely related species (*Placopecten magellanicus*, *Mimachlamys nobilis*, *Mimachlamys varia*, *Mizuhopecten yessoensis* and *Argopecten irradians*) reported to possess *atp8*, lowering the sequence similarity confidence threshold to 60%. Despite this relaxed criterion, the *atp8* gene was still not detected in *A. colbecki*, suggesting a possible absence of this gene in the species. This pattern of the gene loss is consistent with a known variability of animal mitochondrial genomes during the evolutionary history, which typically encode 13 PCGs, but show lineage-specific reductions [41]. The number of PCGs in *A. colbecki* differed from that of *Amusium pleuronectes*, which possesses the complete set of 13 PCGs as observed in most marine bivalves [44].

In addition, the presence of 18 tRNA genes in the mitogenome of *A. colbecki* deviates from the canonical pattern of typically 22 ~ 23 tRNA genes reported in scallops [45,46]. However, mitochondrial genome size and gene composition in bivalves, including scallops, are known to vary considerably due to differences in gene content as well as the presence or absence of specific regions, such as non-coding or barcode-related regions [43]. This variability may also influence the annotation of small or highly divergent genes. To address this, we performed additional annotation analyses and comparative assessments with closely related scallop species (*Placopecten magellanicus*, *Mizuhopecten yessoensis*, and *Chlamys farreri*) using two independent programs (MitoZ v2.3 and Geneious Prime^®^). Despite these efforts, no additional tRNA genes were detected, and the total number of tRNA genes was consistently identified as 18, supporting the present annotation.

The phylogenetic analysis revealed that *A. colbecki* forms a well-supported lineage clearly separated from major scallop genera such as *Argopecten*, *Chlamys*, and *Mimachlamys*. Although the Antarctic scallop is the most closely related to *P. magellanicus*, the long branch length and high bootstrap support values suggest considerable evolutionary divergence between the two species, which is similar as phylogenetic distinction of *Chlamys farreri* and other species belonging to family Pectinidae. The major clades recovered in this study broadly correspond to those reported in previous mitogenome-based phylogenies of Pectinidae [43], although some inconsistencies remain at the subfamily and tribal levels. This result aligns with previous mitogenomic-based phylogenies of Pectinidae [42,43] and likely reflects the species’ long-term evolutionary history and diversification in the Antarctic environment [47]. These results not only enrich the genetic resources available for Antarctic bivalves, but also show the trends consistent with the previous findings, highlighting the value of scallop genomes for understanding ancestral genomic structures and evolutionary history of early bilaterians [48].

The microsatellite analysis identified approximately more than one-hundred thousand perfect SSR loci, with mononucleotide and dinucleotide repeats the most prevalent, which is congruent with the patterns of SSR distributions in other marine bivalves [49]. The newly developed microsatellites, and mtDNA particularly those targeting conserved rRNA regions, can be versatile markers in future studies for population genetics analysis and potentially resource conservation and management, as previously demonstrated in *C. farreri* [49]. Additionally, to evaluate the utility of the seven developed microsatellites, we analyzed the *N*_*A*_, *H*_*O*_, *H*_*E*_, *F*_*IS*_, PIC and HWE for 27–30 individuals of *A. colbecki*. The *N*_*A*_ was 15.3, with an average *H*_*O*_ of 0.6988, *H*_*E*_ of 0.8979, *F*_*IS*_ of 0.225 and PIC of 0.8711. These values indicate higher polymorphism compared to those reported for closely related species. For instance, *Chlamys nobilis* exhibited *H*_*O*_ values ranging from 0.507 to 0.583, *H*_*E*_ between 0.596 and 0.706, and PIC from 0.526 to 0.654 [50]. In *M. varia*, mean *H*_*O*_ and *H*_*E*_ were 0.442 and 0.592 respectively, based on five loci [51]. Nevertheless, further validation across geographically distinct populations is required to confirm the versatility of these microsatellite loci for broad-scale ecological and evolutionary genetic studies. The estimated null allele frequencies were generally low [mean: 0.126 from MICRO-CHECKER; 0.133 from GENEPOP] and fell within ranges commonly reported for highly polymorphic microsatellite markers. In addition, no evidence of genotyping errors such as stuttering or large allele dropout was detected. Therefore, these loci were retained, as the influence of null alleles on downstream population genetic analyses is expected to be minimal.

These markers were developed based on genomic data from *A. colbecki* and are therefore considered specific to *A. colbecki* in this study. However, although cross-species amplification was not evaluated in this study, earlier reports on pectinid bivalves indicate that microsatellite loci frequently exhibit reproducibility among closely related species [43], implying potential broader applicability of these markers beyond *A. colbecki*. The microsatellite markers can also be useful for biomonitoring the ecological status of the *A. colbecki* population, given this species is known to be particularly vulnerable to the climate change as the scallop is incapable of adapting to increasing temperature [18]. By generating the first complete and annotated mitochondrial genome of *A. colbecki*, along with species-specific microsatellite markers, this genomic information provides a baseline for future comparative mitogenomic studies across polar and temperate scallops, contributing to the reconstruction of evolutionary trajectories within the Pectinidae. The availability of mitogenomic and molecular marker resources will facilitate future investigations into population structure, phylogenetic relationships, and adaptive strategies in Antarctic environments.

## Conclusion

This study presents the first complete and annotated mitochondrial genome of the Antarctic scallop *A. colbecki*, along with newly developed seven microsatellite markers. The circular mitogenome, comprising 15,269 bp, includes 12 PCGs, two rRNA genes, and 18 tRNAs, and lacks the *atp8* gene, consistent with patterns observed in other bivalves. We also tested for the polymorphism at 12S and 16S rRNA regions as molecular markers. Phylogenetic analysis based on 11 PCGs (7,053 bp) revealed that *A. colbecki* formed a distinct and well-supported lineage within Pectinidae and was most closely related to *P. magellanicus*. The microsatellite analysis revealed over 144,000 perfect SSR loci, with mononucleotide and dinucleotide repeats being the most common, and yielded primer sets applicable to future studies in population genetics, species identification, and its conservation and biomonitoring. Together, these genomic resources fill a significant gap in our understanding of *A. colbecki* and provide a foundation for further research on the evolutionary history, population structure, and ecological genetics of this ecologically valuable species in the rapidly changing Southern Ocean.

## Supporting information

S1 Table Summary of detailed information on 30 microsatellite markers developed for *Adamussium colbecki.* (DOCX)
