# Supplementary material for: Complete mitochondrial genome and microsatellite marker development of the Antarctic scallop (Adamussium colbecki) for its population genetics analysis
Source: PLoS One. 2026 Jun 15;21(6):e0351123. doi: 10.1371/journal.pone.0351123 (PMC13268180; doi:10.1371/journal.pone.0351123)
Supplement: S1 Table — (DOCX) [file pone.0351123.s001.docx]

**Table S1** Summary of detailed information on 30 microsatellite markers developed for *Adamussium colbecki*.

| **Locus** | | **Primer sequences (5′- 3′)** | **Motif** | **Ta (°C)** | **Product size (bp)** |
| --- | --- | --- | --- | --- | --- |
| ACI-1 | F | CAGTCTTAGAGTTTATTCCC | (GAA)_13_ | 50.27 | 165 |
|  | R | TCAGAACAATCCTATCTGG |  | 50.89 |  |
| ACI-2 | F | AGTAGGAGAGATAGTAATGG | (GA)_20_ | 49.81 | 132 |
|  | R | GACTCGAATGAAAACTAGC |  | 51.37 |  |
| ACI-3 | F | ATTTATTCAGAGAGAGTCCC | (GCA)_13_ | 51.34 | 126 |
|  | R | ATCCTTTCGAGTCTATGG |  | 50.20 |  |
| ACI-4 | F | CTCAAGGTTTTTATCGCG | (CT)_20_ | 51.64 | 109 |
|  | R | GAGAGAGAGAGATACAGC |  | 50.13 |  |
| ACI-5 | F | CTTATATGAAGCCCTTTCC | (GA)_20_ | 50.16 | 153 |
|  | R | GTCACAGTATCATATTTCGG |  | 51.22 |  |
| ACI-6 | F | TGCAGTATAGATAGCTATCG | (GT)_20_ | 51.24 | 182 |
|  | R | CATGGGTAACACAATAACG |  | 51.58 |  |
| ACI-7 | F | CTATGAACCTTGTAAGTAGG | (CTA)_13_ | 49.98 | 183 |
|  | R | GGATAGATGATAATATGCCC |  | 49.95 |  |
| ACI-8 | F | TAATAACACTGGAATACCCC | (TGA)_13_ | 51.59 | 145 |
|  | R | CTATGAAAATTCAGGTTGGG |  | 51.78 |  |
| ACI-9 | F | GATATTCAAAGACACCTCC | (TCA)_13_ | 50.15 | 146 |
|  | R | GAGCTACTGTTATTGTCC |  | 49.37 |  |
| ACI-10 | F | GACCACTTTTATGACTTCC | (TAC)_13_ | 50.72 | 184 |
|  | R | AGTAGTAGTGTAGTAGTAGG |  | 49.17 |  |
| ACI-11 | F | AACTTCTATACCTAGACTCG | (CAG)_12_ | 50.59 | 183 |
|  | R | CTCTCCACTGGAATTACC |  | 51.21 |  |
| ACI-12 | F | CCTGTATATAAAGAGCATCG | (CT)_19_ | 50.69 | 201 |
|  | R | AAGAGAGAGGACTTAATAGG |  | 50.34 |  |
| ACI-13 | F | TCTTTATTTCTCCCCTCC | (CT)_19_ | 49.83 | 103 |
|  | R | CTACCGTGTATTGATATCC |  | 49.47 |  |
| ACI-14 | F | GAGTATTTACGTCATCCG | (GT)_19_ | 49.73 | 142 |
|  | R | ATATCTGGGACAAGAAACC |  | 51.47 |  |
| ACI-15 | F | AATAGACAGAGAGATACAGG | (CAT)_12_ | 50.48 | 124 |
|  | R | GGTTCATATGTATAGTGCC |  | 50.01 |  |
| ACI-16 | F | GTCATAACGGAACATACC | (AT)_18_ | 49.83 | 221 |
|  | R | GTACATAAAACGTAGGTCC |  | 50.29 |  |
| ACI-17＊ | F | GAGTACCTTGTTAAATCGG | (GTT)_12_ | 50.57 | 210 |
|  | R | GATTCGATGACATGTTCC |  | 50.58 |  |
| ACI-18 | F | CAGCGTCCTTAATTATGG | (GT)_19_ | 50.20 | 141 |
|  | R | AGTCTAGATGATACAATCGG |  | 51.32 |  |

| ACI-19＊ | F | ACAAGAAGAAACAGGAGG | (AGG)_12_ | 51.22 | 133 |
| --- | --- | --- | --- | --- | --- |
|  | R | AAGGACAGATACATGTAGG |  | 50.57 |  |
| ACI-20 | F | CAATGAAGTATCTCTGTACC | (GA)_18_ | 50.40 | 196 |
|  | R | CTCTCTCTTTCTCTCTATCC |  | 50.96 |  |
| ACI-21＊ | F | ATGTGTCACTCATGTACC | (CA)_17_ | 51.14 | 135 |
|  | R | TTGAACTATGTATAGGGACC |  | 51.03 |  |
| ACI-22＊ | F | CGGTAGTCCTTTATTTGG | (GAA)_11_ | 49.66 | 150 |
|  | R | GAATACGAGTGTTACTACC |  | 49.72 |  |
| ACI-23＊ | F | CTTCATCATGACATCATCG | (ATC)_11_ | 51.17 | 155 |
|  | R | GGTGATTATATATGACGAGG |  | 50.04 |  |
| ACI-24 | F | GATTCCTAATACATAACCGC | (CT)_17_ | 51.25 | 138 |
|  | R | TTCCAGTTACATGACAGG |  | 51.00 |  |
| ACI-25 | F | CATATATGTCAGCACAAGG | (CA)_17_ | 50.67 | 159 |
|  | R | CTGTACTTGTCATTGTACG |  | 51.00 |  |
| ACI-26 | F | GTATACGTATACTTCAACCG | (AAC)_11_ | 50.75 | 127 |
|  | R | TACTCATAACGTCAGTATGG |  | 51.59 |  |
| ACI-27＊ | F | GTACACAAAAATACCCTCC | (AAG)_11_ | 50.68 | 153 |
|  | R | TGTAGCCAATGTGTTACC |  | 51.77 |  |
| ACI-28 | F | TACTTATCTGTTTCGCGG | (CTA)_11_ | 51.8 | 166 |
|  | R | GTAGTAGTAGTAGCAGTAGC |  | 51.46 |  |
| ACI-29 | F | CATCAGGAAACAGTAATCC | (TGA)_11_ | 50.46 | 184 |
|  | R | CCATCACTCAAGAATTTCC |  | 51.08 |  |
| ACI-30＊ | F | GGATCCACTCTTTTTAGC | (CT)_17_ | 49.95 | 199 |
|  | R | TATAGAGTACTGTAGTGTCG |  | 50.38 |  |

*: Microsatellite markers used in this study are indicated by an asterisk.
